# Minimally Invasive Spinal Arthrodesis in Osteoporotic Population Using a Cannulated and Fenestrated Augmented Screw: Technical Description and Clinical Experience

**DOI:** 10.1155/2012/507826

**Published:** 2012-08-30

**Authors:** Alphonse Lubansu, Michal Rynkowski, Laurence Abeloos, Geoffrey Appelboom, Olivier Dewitte

**Affiliations:** Department of Neurosurgery, ERASME Hospital, Université Libre de Bruxelles, 808 Route de Lennik, 1070 Brussels, Belgium

## Abstract

We describe a percutaneous or minimally invasive approach to apply an augmentation of pedicle fenestrated screws by injection of the PMMA bone cement through the implant and determine the safety and efficiency of this technique in a clinical series of 15 elderly osteoporotic patients. Clinical outcome and the function were assessed using respectively the Visual Analogue Scale (VAS) score and the Oswestry Disability Index (ODI). Peri- and post-operative complications were monitored during a minimum of 2 years of follow-up. Radiographic follow-up was based on plain fluoroscopic control at 3, 6 and 12 months and every year. In this approach, four steps were considered with care: optimal positioning of the screws, correct alignment of the screw heads, waiting time before the injection of cement, fluoroscopic control of the cement injection. Using these precautions, only 2 minor complications occurred. VAS scores and ODI questionnaires showed a statistically significant improvement up to 13.3 months postoperatively. No radiological complications were observed. Based on this experience, PMMA augmentation technique through the novel fenestrated screws provided an effective and long lasting fixation in osteoporotic patients. Applying this procedure through percutaneous or minimally invasive approach under fluoroscopic control seems to be safe.

## 1. Introduction 

Due to the progressive increase of life span and the improvement of the quality of life (QoL) of the elderly, the surgical indications for degenerative and trauma lumbar spine in the aging population is increasing. The current elderly population desires to remain active and resists the acceptance of disability and low back pain. It becomes unavoidable for a spine surgeon to encounter patients with osteoporosis or other decreased bone quality who require spinal decompression and stabilization for degenerative spinal diseases, spinal trauma, infection, tumor, or inflammatory spinal diseases [1–3]. In the young population, the conventional posterior pedicle screw arthrodesis associated with lumbar interbody fusion (LIF) is widely used in spinal surgery to attain rigid stabilization after surgical intervention in situations leading to a progressive mechanical instability [4, 5]. Despite the demonstrated efficacy, some drawbacks are currently reported associated to the extensive soft-tissue dissection that is necessary to facilitate the insertion of the screws and prepare the fusion bed. The muscular incision increases perioperative blood loss, the postoperative pain, and the hospitalization time increases the risk of failed back surgery syndrome [6–9]. As a result, interest has increased for less traumatic surgical approaches that are associated with minimally invasive techniques for pedicle screw placement and LIF, with less postoperative pain and blood loss than conventional open procedures [10].

In the aging population, this interest for minimal invasive techniques is not as evident, probably because the conventional spinal arthrodesis is already considered as challenging [11, 12]. It has been well documented that bone mineral density (BMD) is one of the main factors related to spinal instrumentation failure. The ability of screws to resist pullout from bone is directly related to the BMD [13]. Many potential complications, such as screw loosening, migration, or pullout, compromising the surgical outcome have been described. Several authors reported the efficiency of the augmentation techniques by injecting PMMA into the vertebral body through the pedicle before inserting the screw. However, most pedicle screws are not designed to be used with PMMA. Also, introduction of PMMA through a tapped hole can increase the risk of PMMA leakage through potential breaches that could occur in the pedicular wall during the tapping before screw insertion [14]. To avoid this, a novel-concept cannulated screw with fenestrations in the distal portion of the screw has been designed. After insertion of the screw into the pedicle, cement can be injected and will distribute evenly around the thread of the screw to improve fixation performance [15, 16]. The purpose of this paper is to describe a novel technique using cannulated and fenestrated PMMA augmentable screw in percutaneous and minimally invasive spinal posterior arthrodesis and to report the safety and efficiency of this technique in a prospective patient series.

## 2. Materials and Methods

### 2.1. Study Patients

**Figure 1:**
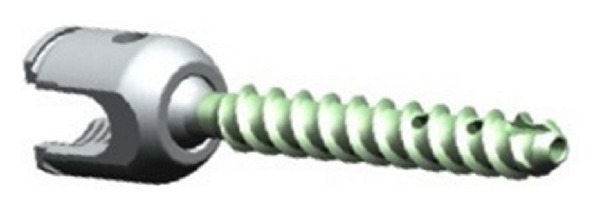
The titanium Expedium fenestrated screw (VIPER MIS Spine System, DePuy Spine, Johnson & Johnson) is a polyaxial, fully cannulated with six fenestrations in the grooves of the distal portion of the thread and an opening at the distal tip.

A consecutive prospective series of 15 osteoporotic patients operated on between March 2010 and July 2011 (12 female, 3 male, mean age 71.2 years (60–88)) with osteoporotic compression/burst fracture (4 patients), degenerative spondylolisthesis (5 patients), and spinal and/or foraminal stenosis (6 patients) underwent MIS posterior pedicle arthrodesis with or without interbody fusion with PMMA cement augmentation of pedicle screws. All patients were included in this study based on the results of a DEXA bone mineral density examination showing osteopenia to severe osteoporosis according to the WHO criteria. The mean T score was −2.7 (−2.1 to −4.1).  Figure 1 shows the new model of cannulated and fenestrated pedicle screw featuring fenestrations that allows cement injection through the implant. Expedium fenestrated screws (DePuy Spine, Johnson & Johnson) was used in all cases. 

Inclusion criteria were as follows: (1) patient over 60 years of age; (2) demonstration by DEXA bone mineral density examination of osteopenia to severe osteoporosis according to the WHO criteria; (3) evidence of spinal trauma, degenerative or deformative spinal disorders with an indication of stabilization and realignment of the thoracolumbar or lumbar spine. Patients were excluded from the study in case of (1) previous history of spinal infection; (2) spondylolisthesis > grade III; (3) severely increased risk for surgery under general anaesthesia due to cardiovascular, pulmonary, or other concomitant diseases. The mean follow-up period was 13,3 months (6 to 24 months).  Table 1 shows the demographic characteristics of the included patients and their clinical data. All patients were evaluated using CT scan or MRI to define the surgical indication and to measure the pedicle diameter and length prior to surgery.

In all cases, preoperative clinical data were collected: pain intensity was evaluated by the VAS and the function was assessed by the ODI [17].

### 2.2. Surgical Technique and Instrument

All the patients were operated under general anaesthesia. A “flash” dose of antibiotic (cephalosporin) was injected intravenously 1/2 hour before the incision and renewed once the surgery lasted longer than 3 hours. 

Patients were placed in a prone position on a radiolucent standard operating table with chest and pelvis supported to gain correction of kyphotic deformity when needed. Conventional C-arm fluoroscopy was used for the entire procedure (Arcadis; Siemens; Munich, Germany). 

**Figure 2:**
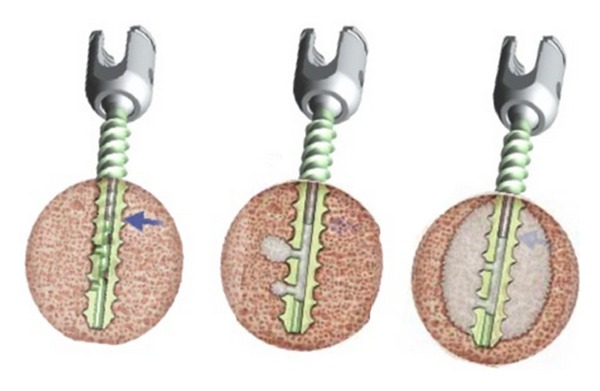
The cement is extruded through the fenestrations to fill the spaces inside the osteoporotic cancellous bone. The cement used is PMMA bone cement (Vertebroplastic, DePuy Spine, Johnson & Johnson).

The novel pedicle screw used in this series was the titanium Expedium fenestrated screw (VIPER MIS Spine System, DePuy Spine, Johnson & Johnson) which is a polyaxial and fully cannulated screw with six fenestrations in the grooves of the distal portion of the thread and an opening at the distal tip (Figure 1). A specific delivery system, including alignment guides, cement delivery cannula for use with the V-MAX Mixing, and delivery system, was used to inject the cement under controlled pressure through the cement cannula. PMMA bone cement (Vertebroplastic, DePuy Spine, Johnson & Johnson) (Figure 2) was extruded through the fenestrations to fill the spaces inside the osteoporotic cancellous bone. 

### 2.3. Operative Steps

Under exact fluoroscopic antero-posterior view of the vertebral body, the projections of the target pedicles are identified and drawn on the skin. Depending on the surgical plan, a pure bilateral percutaneous pedicle screw arthrodesis or a combination of unilateral percutaneous associated with a contralateral mini-open (modified Wiltse [5]) can be realised.

For the pure percutaneous fenestrated screw placement, a skin incision is made 10 to 20 mm lateral to the pedicle's upper quadrant projection. The thoracolumbar fascia is split and a targeting needle is used to introduce a K-wire guide inside the pedicle. Successive AP and lateral fluoroscopic images are taken to accurately identify the pedicle entry point, the optimal position of the needle at the posterior wall of the vertebral body, and the good alignment of the needle with the desired screw trajectory. A K-wire guide is then placed in the needle and advanced in the two-thirds of the vertebral body. We placed pedicle K-wire guides in all target pedicles as during the first step of the procedure.

**Figure 3:**
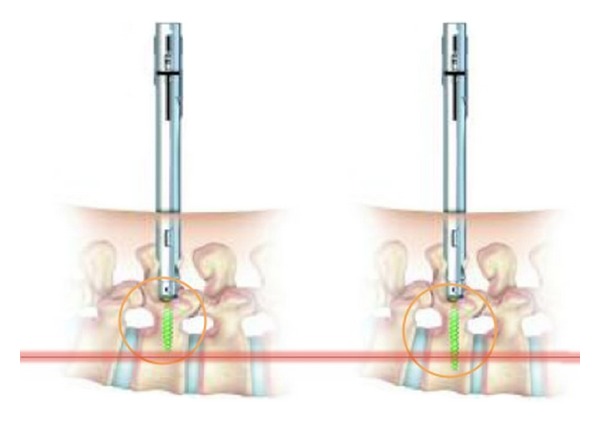
When fenestrated screw is placed through the percutaneous or miniopen approach, the length of screw is important because of the risk of extravasation of PMMA bone cement. An optimal alignment with the pedicle is recommended. Position of the holes must be located as far as possible from the posterior wall.

**Figure 4:**
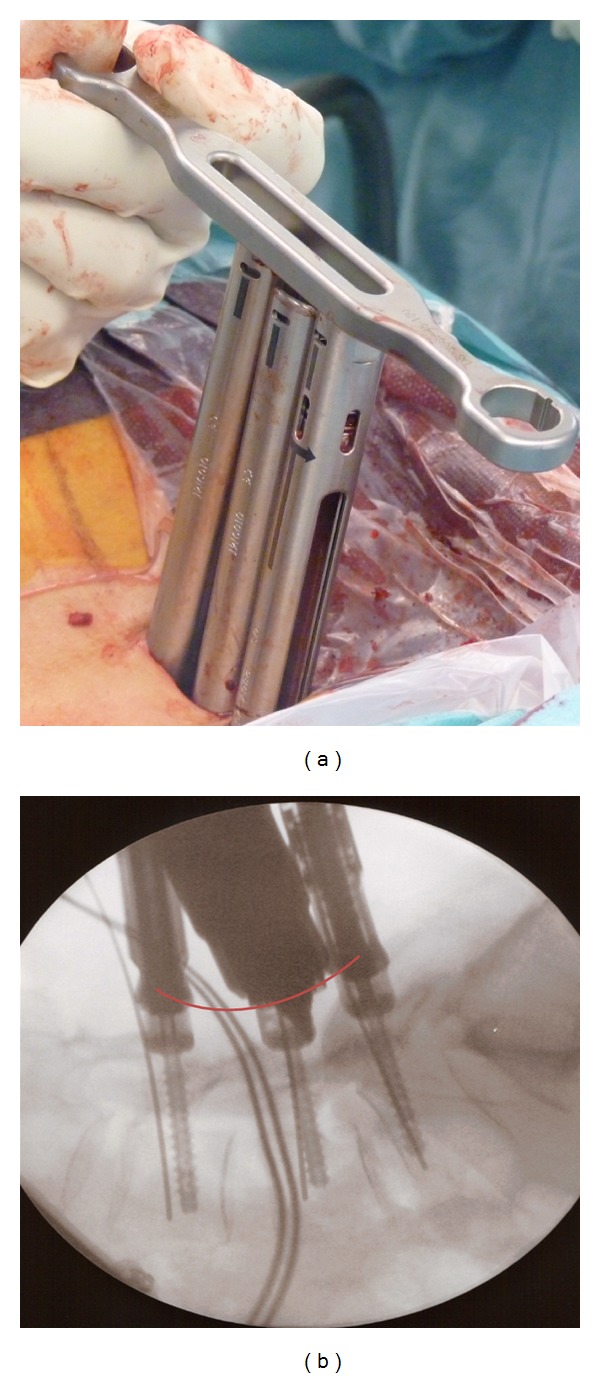
The optimal alignment of the heads of the screws is important. He can be controlled at the top of the screw extenders (a) or on a lateral fluoroscopic view (b). When all the fenestrated screws are optimally placed, we suggest testing the unconstraint placement of the rod to avoid positioning issues during the definitive rod placement after cement injection.

Dilators of progressively larger sizes are used to create the working channel by dilating the muscle tissue. A tap (undersized to the screw) is advanced over the K-wire to prepare the screw placement. The fenestrated screw is inserted into the pedicle guide over the K-wire with a selected length of screw and the position of the holes, located as far as possible from the posterior wall to prevent possible PMMA leakage into the spinal canal (Figure 3). Each fenestrated screw is attached to an extender sleeve. When all the fenestrated screws are optimally placed, we suggest to make a trial of the unconstraint placement of the rod to avoid positioning issues during the definitive rod placement after cement injection. After PMMA augmentation, alteration of the screw position is no longer possible (Figures 4(a) and 4(b)). 

The rod insertion is done through one of the percutaneous skin incisions under the muscular fascia. After correct rod placement, the closure tops are tested. 

When a central canal decompression or a transforaminal interbody fusion (TLIF) is planned, the described percutaneous procedure is done unilaterally along with a mini-open approach as illustrated by Holly et al. [18] using a multiple blade retractor before the placement of the pedicle screws. The bone graft used for the TLIF or for the posterolateral fusion is a mixture of (1) autologous local bone shavings, (2) allograft from cadaver bone bank, and (3) bone marrow aspirated from the posterior iliac crest. When the canal recalibration or the placement of interbody cage filled with bone graft is done, the fenestrated screws are placed over the K-wire using the same steps as described before.

**Figure 5:**
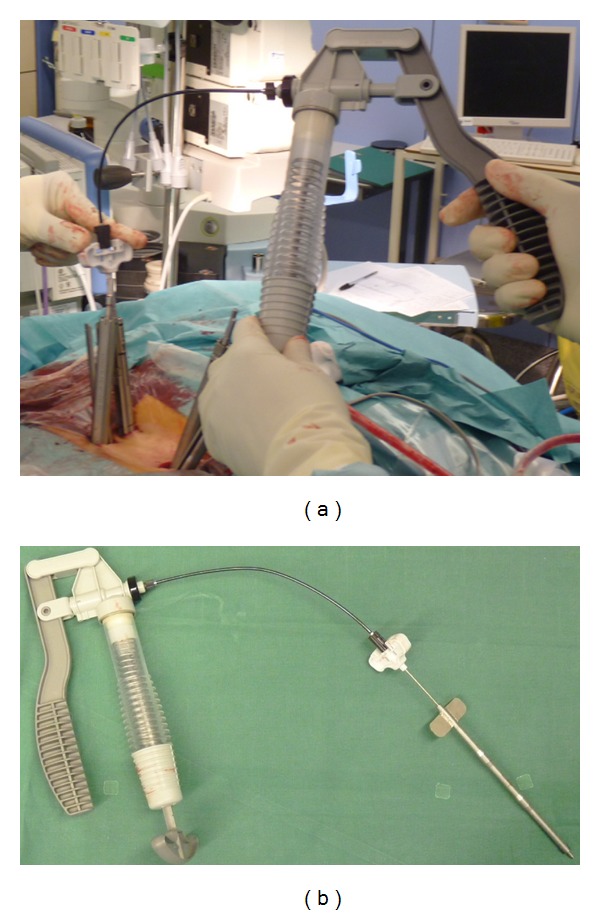
The screw and the cement delivery system are connected using a specifically designed connector. The PMMA bone cement is delivered through the cement cannula placed within the cannulation of the fenestrated screws under continuous image intensifier visualisation.

**Figure 6:**
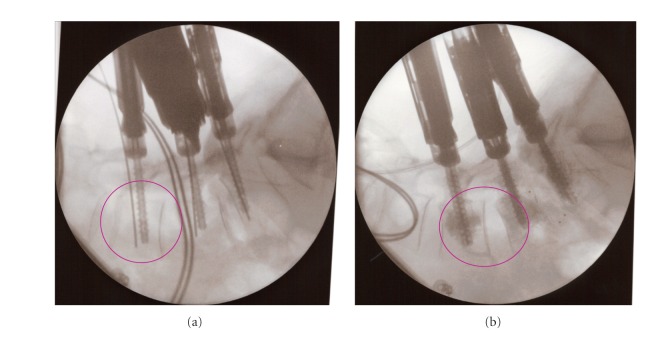
Injection must be done under fluoroscopic control to immediately stop the injection in case of cement extravasation.

The screw and the cement delivery system are connected using a specifically designed connector. The PMMA bone cement is delivered through the cement cannula placed within the cannulation of the fenestrated screws under continuous image intensifier visualization (Figure 5). The amount of cement injected into each screw varies from 1.5 to 3 mL. We experienced that the ideal amount of cement to inject was 2 mL. To prevent cement leakage, the injection was done in a higher viscosity state (started 5 minutes after mixing). The cement injection was stopped in case of any leakage of cement (anterior, posterior, or into an adjacent disc) (Figure 6).

### 2.4. Perioperative Data

A total of 78 fenestrated screws were implanted (min 4; max 10 per patient), in combination with standard cannulated Viper screws (when sacral screws were placed bicortically). The operative blood loss, duration, and complications were monitored. PMMA extravasations were documented if occurred during the injection procedure.

### 2.5. Postoperative Care

Depending on patient's clinical situation, patients were allowed to ambulate with protected thoracolumbar-sacral orthosis or lumbar-sacral orthosis 48 hours after surgery. The orthosis was maintained until the confirmation of the optimal screw placement and the absence of radiological complications on a postoperative thoracolumbar CT scan. All patients were followed up at the outpatient department at 3, 6, and 12 months, and then regularly every year. 

The followup was clinically documented using the ODI [17]. In addition, the patients had to assess their radicular and low back pain on a 10 cm VAS between 0 (no pain) and 10 (maximal pain). The preoperative and postoperative VAS and ODI were compared with a paired *t* test. Statistical significance level was defined as *P* < 0.05.

### 2.6. Radiological Outcome Assessments

A radiographic evaluation was also performed at each followup based on standard radiographs for signs of screw loosening, loss of sagittal alignment (kyphosis), and screw migration. Optimal intervertebral or posterolateral fusion was considered on radiographs if (1) presence of bone bringing inside and/or around the cage and (2) absence of radiolucency lines around screws or cages were noted at 12-month follow-up radiographic control.

## 3. Results

The clinical results are summarized in Table 1. All 15 patients had osteoporosis with a DEXA bone mineral density examination showing moderate to severe osteoporosis. Seventy-eight cement-augmented fenestrated screws were placed on a total of 82 screws (4 bicortical standard screws were placed in S1 without injection of PMMA). The surgical indication was degenerative in 73.3% (11/15 patients) and osteoporotic burst fracture in 26.6% (4/15 patients). Short segment fusions were performed in 3 patients to reduce operative times and minimize potential morbidity. Comorbidity factors were found in 12/15 of the patients. Medical history of previous spinal surgery was noted in 6/15 patients (2 disc herniation surgeries, 2 decompression laminectomies, 2 arthrodesis). 5/15 of the patients were smokers. The surgical procedure consisted of percutaneous stabilisation using the augmented fenestrated screws in 6 cases and an unilateral percutaneous stabilisation associated with a contralateral TLIF or bone graft placement through a miniaccess approach in 9 patients. The mean operative time was 165 min⁡± 54.4 (range, 80–275 min), and the mean perioperative blood loss was 261.4 mL ± 195 (range, 30–600 mL). The mean cement injection per pedicle was 2.02 mL ± 0.56 (range, 1.5–3.0 mL). The injection of PMMA was done in a minimum of 5 minutes after mixing to obtain a high viscosity consistency of the cement. Despite this waiting time, PMMA asymptomatic extravasations were observed in 5/15 patients. PMMA extravasations were posterior towards the spinal canal (*n* = 2), in the intervertebral disc (*n* = 1), and into the external venous plexus (*n* = 2). PMMA extravasations were noted in 4 of the 78 fenestrated screws placed (5% of screws). There were no cases of severe morbidity post-operatively (no death, no myocardial infarction, no pulmonary emboli, or intraoperative hypotension). Two postoperative complications related to the procedure were noted: and one S1 screw misplacement associated with a nerve radiculitis (no cement injected through this screw), one subcutaneous infection with multisensible *staphylococcus epidermidis *treated with 2 weeks of antibiotherapy. Patients were observed at regular intervals for a maximum of 2 years. The mean follow-up period in this study was 13.3 months (range, 6–24 months). At the end of follow-up period we noted no construct failure, no screw fractures, no loss of correction, or screw pullout. 

**Figure 7:**
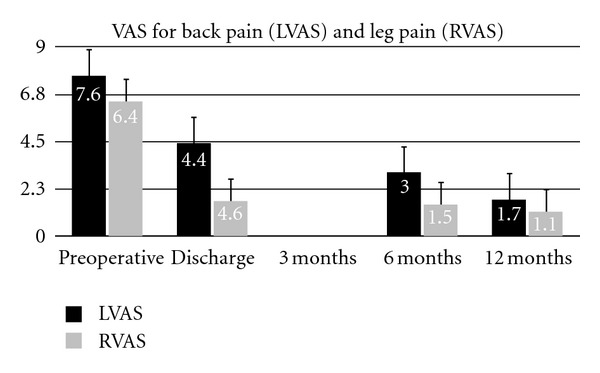
Clinical outcomes preoperatively and over 1 year postoperative followup. Results are expressed as mean scores ± Standard deviation at each time point. LVAS: Low back visual analogue score (1–10) of pain, RVAS: radicular VAS.

Based on the VAS for back pain and leg pain, pain intensity was significantly improved at discharge, 6 months and 1-year followup (Table 2), The back function evaluated by ODI score showed significantly improvement when compared between preoperative and discharge period including 6-month and 1-year followup (Figure 7). Based on the 1-year follow-up Rx control, the fusion was considered as completed in all cases where TLIF or posterolateral bone graft were placed (7 patients). In fracture cases, no bone graft was placed. Nevertheless, the burst fracture was consolidated in all patients. In patients 7 and 10, despite the absence of interbody bone grafting, a spontaneous progressive interbody fusion was noted. 

### 3.1. Illustrative Case

#### 3.1.1. Presentation and Examination

**Figure 8:**
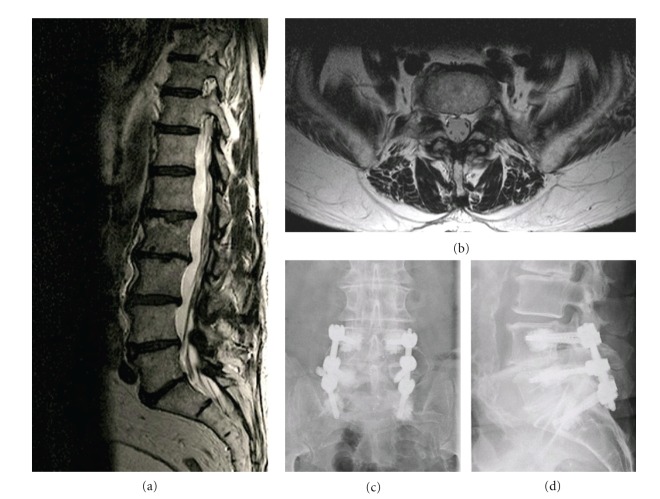
Illustrative Case number 9. Radiological studies obtained in a 83-year-old man. Sagittal (a) and axial (b) T2-weighted magnetic resonance images of the lumbar spine, showing narrowing of the spinal canal at L4–5 and L5-S1 and bilateral foraminal stenosis. (c and d): AP and lateral radiograph at 12 months postoperative demonstrating the proper position of the screws and cages, and the absence of implant-related complication.

This 83-year-old woman presented with more than 5-year history of low back pain, more significant left buttock, lateral calf, and foot pain, as well as intermittent claudication. The pain increased while walking, but the pain was reduced when sitting or bending forward. On physical examination, hypoesthesia was noted in the L5 dermatome bilaterally. The pinprick sensation was decreased in the L-5 dermatome and no motor weakness was detected. The deep tendon reflexes were reduced in the left leg and the straight leg-raising sign was negative. Electromyography examination suggested left L-4 and L-5 radiculopathy. Sagittal MR imaging revealed L4-L5 and L5-S1 discopathy and disc herniation, spinal stenosis, and bilateral foraminal stenosis more marked at the level (Figures 8(a) and 8(b)).

#### 3.1.2. Surgical Procedure

A right percutaneous arthrodesis with augmented fenestrated pedicle screws in L4-L5 and S1 combined with a contralateral minimal access total L4-5 and L5-S1 facetectomy and TLIF (with interbody cages filled with a mixed allograft and autologous bone marrow) was performed. A recalibration of the canal was performed through the unilateral miniaccess. A minimal asymptomatic paravertebral lateral extravasation of PMMA was noted.

#### 3.1.3. Postoperative Course

The patient's case was reviewed at 12 months postoperatively. Control lumbar spine radiography confirmed the stability of the fusion, as well as the absence of hardware failure (Figures 8(c) and 8(d)). Clinically, the patient noted a significant reduction of the preoperative pain and a walking perimeter objectively increased.

## 4. Discussion

In recent years, minimally invasive surgical techniques to perform spinal stabilization have gained in popularity due to the demonstration of reduced perioperative muscular damage, blood loss, postoperative pain, and rehabilitation time [19–24]. Reported as safe and effective in the normal population, those techniques have been referred to the aging population with poor bone quality as a contraindication. Indeed, in elderly patients, the conventional open procedure of arthrodesis using posterior pedicle screws are considered as a challenge. Many complications have been reported and correlated with decreasing bone mineral density [11–13].

Carreon et al. [25] reported after lumbar arthrodesis that at least 1 major complication occurred in 21% and at least 1 minor complication in 70% of elderly patients. Okuda et al. [26] reported 16% of postoperative complications in elderly patients after PLIF with pedicle screw placement. Dong et al. [27] was the first to analyse the potential interest of a mini-open TLIF approach for single-level instrumentation degenerative spondylolisthesis and stenosis with instability in elderly adults and reported a good clinical and radiological outcome associated with a low rate (7.4%) of minor complications. Nevertheless, more recently, in a larger retrospective series, Lee and Fessler [28] reported an overall rate of perioperative and postoperative complications of 20% without significant difference comparing with a young population. Karikari et al. [29] retrospectively reviewed their series of elderly patients who underwent minimally invasive lumbar interbody fusion and found an overall rate of major complications of 7.4% and a total complication rate of 32.4%. Unfortunately, they failed to distinguish posterior and lateral based approaches in their analysis of minimally invasive lumbar interbody fusion, limiting the applicability of their results. The mean followup in this study was 14.7 months. None of the above-mentioned studies reported their fusion rate at the end of followup. 

In our study, we firstly describe the different surgical steps of the percutaneous (or through an miniopen access) placement of a novel cannulated and fenestrated screw designed to allow the injection of a PMMA bone cement through the implant following the optimal positioning of the screw inside the pedicle and the vertebral body. This augmentation technique was already reported in conventional open approach to reduce the complications related to the bone-implant interface (pullout of screw, implant fracture) [15, 30, 31] but never through a percutaneous or minimally invasive approach. 

Various studies demonstrate that PMMA bone cement used to augment screws in osteoporotic bone enhance the screw-bone fixation by 49 to 162% [32, 33].

Fransen [15] suggests that the direct injection of cement through the screw can provide to the implant an immediate improved anchoring and that the filling of the vertebral body (VB) can decrease the risk of compression fractures at the treated levels. This technique can also be used in association with kyphoplasty of the fractured VB, allowing correction of the kyphosis with short-length constructs [15]. This augmentation technique also reduces the risk of extravasation of injected cement. Cement extravasation was observed when a screw was inserted inside a screw hole prefilled with cement [34]. In 2005, Yazu et al. published an experimental study conducted on osteoporotic cadaveric vertebrae and compared the performance of fenestrated screws with traditional screws without cement augmentation. Yazu et al. concluded that cement injection could be controlled more accurately using fenestrated screws, reducing the risk of leakage into the canal and/or foramina [35]. Recently, Amendola et al. [36] confirmed in a prospective cohort series of 21 patients that fenestrated screws for cement augmentation provided effective and long lasting fixation in patients with poor bone quality due to osteoporosis or tumors. No cases of loosening were recorded after a mean followup of 36 months. In our series, no major complication was reported. Two patients developed minor complications (1 transient radiculitis and 1 subcutaneous infection). There were no late complications after 1 year of follow-up. To the best of our knowledge, this paper is the first report of a cement augmentation technique of pedicle screws through a percutaneous or minimally invasive approach. In this technique, three steps must be considered as critical. First, the positioning of the screw must be perfectly aligned with the pedicle with a good convergent trajectory. No fractures of the anterior and lateral cortex of vertebral body can be tolerated to avoid cement extrusion in the retroperitoneal space. Secondly, to avoid breakage of the cement bridges between the screw and the bone, a definitive positioning of the screw must be controlled and the fixation system should be locked and the rods tested in position before injecting. No torsion movement should be applied to the screw after injecting the cement. Thirdly, the cement injection started only when the cement reached a high viscosity state to avoid extravasation. Finally, cement injection must be performed under continuous fluoroscopic imaging to provide immediate visual feedback and control to stop the injection in case of any sign of extravasation. Despite this caution technique, we report 33% of radiological PMMA cement extravasation; however, none were symptomatic. As it has been demonstrated that the pullout strength did not significantly increase with the volume of cement injected over a range of 1.5 mL [37, 38], we suggest to inject maximum 1.5 to 3.0 mL of cement per screw. In this serie, the mean volume of injection was 2.02 mL ± 0.56 per screw. In Table 3, we summarized the suggested tips to prevent PMMA cement extravasations. 

Similarly, as described for the young population, in our elderly population the MIS procedures were associated with a low rate of peri- and postoperative blood loss, postoperative pain, hospital stay, and recovery time. The clinical state of the patients was significantly improved and this improvement was maintained during the short followup of this clinical series. The radiological outcome was also excellent in all cases. Paré et al. [38] tested the biomechanical removal of cement augmented pedicle screws in cadaver spines. In the majority of screws, the removal was easy; in two removals, some bone cement remained attached to the screws and created secondary fractures to the pedicle. They suggested to control this potential removal in a real clinical situation under fluoroscopic control to prevent inadvertent damage on pedicle.

In this primary experience, a systematic amount of radiation exposure was not available. Nevertheless, we highly suggest to monitor the annual radiation exposure of surgeons and to apply all recommendations to reduce this exposure. The need for lead shielding cannot be overstated. The use of thyroid shielding, leaded glasses, and radiation attenuation gloves is absolute. Despite the interest of this study, a longer followup would be important in order to consider this novel technique as an effective one. A controlled randomized study could be suggested. 

## 5. Conclusions

The PMMA augmentation technique of fenestrated pedicle screws is a safe technique to increase the pullout strength of screws placed in osteoporotic spines. This is the first clinical report of this augmentation technique through a percutaneous and/or a minimally invasive approach. We can confirm the safety and efficacy of this technique to prevent the short-time complications as described in performing arthrodesis in aging populations. The ultimate safety of using this technique in this vulnerable population needs of course to be confirmed in a larger series with a longer followup. The risk associated to PMMA extravasation remains the critical part of this technique. At the start of injecting the high viscosity consistency of the cement, the strict usage of fluoroscopic control should be used to immediately detect any radiological sign of extravasation to prevent severe complications.

## Figures and Tables

**Table 1:** Clinical data of patients undergoing fenestrated pedicle screw augmentation^a^ through minimally invasive approach.

Patient number	Sex/Age (yrs)	Preoperative diagnosis	Comorbidity factors	Surgical history	T/	Fixation levels	Surgical procedure	Bone graft	Complication	PMMA extravasation (number of screw related)	FU (mo)
(1)	F/60	Degenerative discopathy, stenosis	None	Disc herniation	N	L3-S1	Percutaneous augmented FS + miniaccess TLIF	Autologous + allograft (Ant.)	Transient Radiculitis, Screw misplacement	None	24
(2)	F/61	L4 Burst fracture	BMI > 30, AVC, viral hepatitis, depression	N	Y	L3-S1	Percutaneous augmented FS	None	None	None	18
(3)	F/74	L1 Burst fracture	NH Lymphoma, cerebral hematoma, dementia, ethylism	N	N	D12-L2	Percutaneous augmented FS	None	None	None	12
(4)	F/73	L1 Burst fracture	Angor, ethylism, depression, arteritis MI	Disc herniation	Y	D12-L3	Percutaneous augmented FS	None	None	None	12
(5)	F/66	Degenerative spondylolisthesis, disc herniation	Nephrectomy, hypertension, pace maker, epidural fibrosis	Arthrodesis	N	L4-L5	Percutaneous augmented FS + miniaccess TLIF	Autologous + allograft (Ant.)	None	None	21
(6)	F/71	Degenerative discopathy, stenosis	None	N	Y	L3-S1	Percutaneous augmented FS + miniaccess TLIF	Autologous + allograft (Ant.)	None	None	12
(7)	M/70	Degenerative discopathy, stenosis	Hypothyroidism	N	N	L2-L3	Percutaneous augmented FS	None	None	Lateral external venous plexus, asymptomatic (1)	12
(8)	F/75	Degenerative discopathy, stenosis	Rheumatoid arthritis	N	N	L4-L5	Percutaneous augmented FS + miniaccess TLIF	Autologous + allograft (Ant.)	None	Posterior leakage asymptomatic (none)	14
(9)	M/83	Degenerative discopathy, stenosis	HTA, ischemic cardiomyopathy, prostate adenoma, atrial fibrillation, renal insufficiency	N	N	L4-S1	Percutaneous augmented FS + miniaccess TLIF	Autologous + allograft (Ant.)	None	Lateral external venous plexus, asymptomatic (1)	16
(10)	F/71	Degenerative discopathy, stenosis	Depression, hypertension	Laminectomy	Y	L4-S1	Percutaneous augmented FS	None	None	None	17
(11)	F/75	Degenerative spondylolysthesis, stenosis	BMI > 25, type 2 diabetes, hypertension, parkinson	N	N	L3-L5	Percutaneous augmented FS + miniaccess TLIF	Autologous + allograft (Ant.)	None	None	13
(12)	F/58	L4 burst fracture	Hypertension, depression	N	Y	L3-S1	Percutaneous augmented FS	None	None	None	10
(13)	M/78	Degenerative discopathy, stenosis	Obesity, hypertension, type 2 diabetes	N	N	L3-S1	Percutaneous augmented FS + miniaccess for bone graft	Autologous + allograft (Post Lat.)	Subcutaneous infection	Posterior leakage asymptomatic (1)	8
(14)	F/72	Degenerative spondylolysthesis, stenosis	BMI > 30, cerebral aneurysm embolized	Laminectomy	N	L2-L4	Percutaneous augmented FS + miniaccess for bone graft	Autologous + allograft (Post Lat.)	None	None	12
(15)	F/68	Degenerative spondylolysthesis, stenosis	None	Arthrodesis	N	L2-S1	Percutaneous augmented FS + miniaccess for bone graft	Autologous + allograft (Post Lat.)	None	Intradiscal extravasation, asymptomatic (1)	6

Total	12F/3M, 71.2 yrs^b^					78 augmented screws			2 out of 15, 13,3%	5 out of 15, 30% (no symptomatic)	13,3 mo mean FU

^a^PMMA: polymethylmethacrylate; ^b^mean age (yrs); T/: tobacco; CA: carcinoma; BMI: body mass index; FS: fenestrated screws; TLIF: transforaminal lumbar interbody fusion; FU: followup; ant.: Interbody anterior bone graft; Post Lat.: posterolateral bone graft.

**Table 2:** Means LVAS, RVAS, and ODI scores at preoperative, discharge, 6 months and 1-year postoperative.

	Lumbar VAS (LVAS)	Radicular (VAS)	ODI
Preoperative	7.6 ± 1.8	6.4 ± 1.7	34.1 ± 11.6
Discharge	4.4 ± 1.9*	1.6 ± 1.4*	
6 months postop.	3.0 ± 2.6*	1.5 ± 1.8*	16.2 ± 8.8*
1-year postop.	1.7 ± 2.9*	1.1 ± 1.5*	14.9 ± 9.7*

*P < 0.01 when compared to same score at preoperative.

**Table 3:** Tips suggested to prevent PMMA cement extravasations.

(1) An optimal positioning of the fenestrated pedicle screws is crucial	
(2) Screws must be placed in the middle of the pedicle to avoid cortical breaches	
(3) A good preoperative CT planning is recommended to select the correct diameter of screws	
(4) No injection if breaches suspected or if bicortical screw fixation	
(5) Start injection of cement when the high viscosity is obtained	
(6) Make injection under fluoroscopic control	
(7) Prefer the used of a controlled delivery system so as the V-MAX to be able to stop immediately the injection	
(8) Avoid to inject high volume of cement (we suggest 1.5 to 3 mL/screw)	
